# Impacts of Anthropogenic Activities and Climate Change on the Distribution Ranges of Five *Tragopan* Birds in China

**DOI:** 10.3390/biology15090713

**Published:** 2026-04-30

**Authors:** Jiming Cheng, Chao Zhang, Xingfu Yan, Xinyue Chen, Yingqun Feng, Furong Cai, Hongjin Yan, Shuqi Liu, Yonghong Luo

**Affiliations:** 1 School of Biological Science and Engineering, North Minzu University, Yinchuan 750021, China; 2025508@nmu.edu.cn (J.C.); zhangchao2023@mails.ccnu.edu.cn (C.Z.); 18695256358@163.com (F.C.); y2604919305@163.com (H.Y.); 15648140626@163.com (S.L.); 2 Key Laboratory of Ecological Protection of Agro-Pastoral Ecotones in the Yellow River Basin, National Ethnic Affairs Commission of the People’s Republic of China, Yinchuan 750021, China; 3 School of Life Sciences, Central China Normal University, Wuhan 430079, China; yyyxchen@163.com (X.C.); fyq0016094@163.com (Y.F.)

**Keywords:** climate change, human activities, land use change, *Tragopan* birds, species distribution models

## Abstract

Anthropogenic activities and environmental changes have exerted an increasingly high impact on the habitats of wild animals, especially endangered species. Researchers have paid attention to the effects of future climate change on wildlife habitats. However, the impact of climate change on the suitable habitats of *Tragopan* birds has rarely been reported. Here, we used the Maxent model to assess the influence of climate change on the geographical distribution of five *Tragopan* species. The results showed that the SSP585 scenario projected relatively favorable conditions, with the total area of suitable habitats expected to show an overall increasing trend over time. Centroid analysis revealed that the centroid gradually shifts toward lower latitudes and elevations due to climate warming. Environmental factor analysis showed that human-induced factors (particularly land use) are the main determinants affecting the habitat suitability of *Tragopan* birds. Notably, a comparison between dispersal velocity and biological velocity showed that despite the predicted gradual expansion of habitat area, *Tragopan* birds may be difficult to expand into the newly suitable habitat regions. We further emphasize that establishing ecological corridors and setting up new protected areas will have a more significant impact on conserving the *Tragopan* birds.

## Simple Summary

Human activities and climate change strongly affect wildlife habitats, including *Tragopan* birds (several of which are threatened). Using the Maxent model, we evaluated climate change’s impact on five *Tragopan* species, currently concentrated in southwest and southeast coastal China (Sichuan, Chongqing, and Fujian are optimal). Under the SSP585 scenario, their suitable habitats are projected to expand overall, with centroids shifting to slightly lower latitudes and lower elevations within certain regions. This pattern may be explained by local topoclimatic heterogeneity or the release of non-climatic constraints (e.g., land use changes), although this contrasts with the general expectation of upward and poleward shifts under warming. Land use is the key determinant of habitat suitability. Despite habitat expansion, colonization of new suitable areas by *Tragopan* birds is likely to be limited or potentially constrained due to mismatched dispersal and biological velocities. We highlight the importance of ecological corridors and new protected areas for their conservation.

## 1. Introduction

Climate change driven by human activities has gradually become one of the important factors affecting species survival and distribution [1,2]. Since the period 1850–1900, the global average surface temperature has increased by approximately 1.1 °C [3]. Furthermore, in recent years, significant changes have occurred in temperature and precipitation patterns, with frequent extreme temperatures and droughts, and climate change poses a severe threat to biodiversity [4,5,6]. Previous studies have shown that rising temperatures may lead to a decline in species diversity [7]. Drought can reduce the survival capacity of species, thereby affecting their regional distribution patterns [8]. Through direct or indirect effects (e.g., habitat degradation), climate change impacts species survival, reproduction, and geographical distribution, which in turn affects the health and stability of ecosystems [1,9]. In particular, the impact of climate change on endangered species is more pronounced, easily leading to the regional extinction of endangered species [2,10]. Therefore, exploring the impact of climate change on species distribution is of great importance for animal conservation, especially for endangered species.

Land use changes (e.g., road construction, industrial and agricultural activities, urbanization) can alter habitats, affecting species survival and distribution, which may ultimately lead to reduced population sizes and shrinking distribution areas [11,12]. Many studies have shown that human activities that induce urbanization and habitat fragmentation affect the survival of many wild animals, particularly endangered species [2,13,14]. Information on the spatiotemporal dynamics of habitats is crucial for the effective conservation of endangered wild species and has become the focus of global biodiversity and conservation [10,15].

When climate and land use changes lead to a gradual reduction in species distribution ranges, species can effectively avoid the risk of extinction if they can successfully migrate to suitable habitats [16]. Some studies have pointed out that the distribution ranges of terrestrial species have moved toward higher latitudes by an average of 6 km per decade over the past 30 years [17]. However, this general pattern is not universal; local exceptions can occur, especially in mountain regions or where land use change dominates over climate forcing. When species are unable to migrate to suitable habitats, they face severe survival challenges [18]. The impacts of climate and land use changes on species distribution are a key research focus. A central question is whether species can successfully migrate to suitable habitats, which is critical for understanding their adaptation to these changes.

Predicting species distribution can provide a certain scientific basis for species conservation. Species distribution models (SDMs) predict which areas in other regions have suitable environmental conditions for a species to survive by analyzing the species’ known distribution data and corresponding environmental variable data, thereby effectively predicting the species’ potential distribution range [19]. The maximum entropy model (Maxent) is a commonly used method in species distribution modeling (SDM). This method can perform relatively well with presence-only data and small sample sizes, provided that sampling bias is minimized and model settings (e.g., regularization, feature classes) are appropriately chosen [20]; however, its performance is sensitive to data quality, environmental predictor selection, and parameter tuning. Additionally, the maximum entropy model has the advantages of fast calculation speed and flexible operation [14,21]. Therefore, it is widely used in studies on changes in species’ geographical distribution patterns under human disturbance and climate change scenarios [14,21]. For example, the Maxent model has been used to assess the impact of future climate, human activities, and land use changes on the habitat distribution of animal groups such as mammals [22], primates [2], and birds [23]. Furthermore, the Maxent model has been successfully applied to habitat assessment of endangered birds such as hornbills [14] and crested ibises (*Nipponia nippon*) [24]. However, research on the species distribution of rare and protected birds (e.g., the *Tragopan* birds) remains relatively scarce.

**Figure 1:**
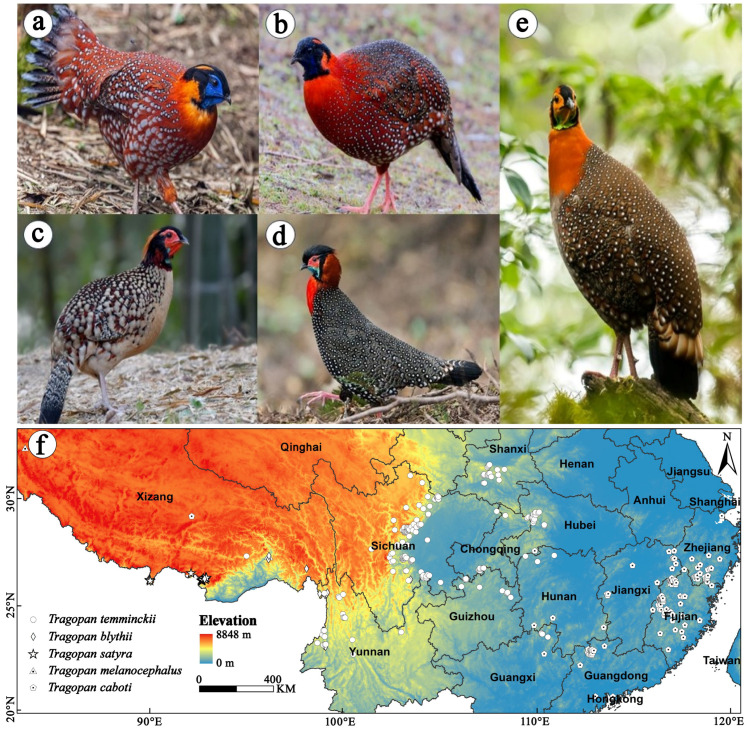
Map of occurrence points of *Tragopan* species in China, with elevation shown. (**a**) *Tragopan temminckii*. (**b**) *Tragopan satyra*. (**c**) *Tragopan caboti*. (**d**) *Tragopan melanocephalus*. (**e**) *Tragopan blythii*. (**f**) Locations of 315 occurrence records (shown by white dots) of *Tragopan* species in China. Bird images are cited from https://ebird.org/home.

The *Tragopan* genus (Phasianidae) is a group of montane forest pheasants endemic to East Asia and the Himalayan region, comprising five species: *T. temminckii*, *T. satyra*, *T. caboti*, *T. melanocephalus*, and *T. blythii* (Figure 1). These birds are regarded as “bio-indicators” of forest health due to their sensitivity to habitat disturbance and fragmentation, as well as their ecological functions [25]. They rely on intact mountain broadleaved forests and coniferous–broadleaved mixed forests and maintain ecosystem balance through seed dispersal and insect regulation [26,27].

According to the International Union for Conservation of Nature (IUCN), the global conservation status of the five *Tragopan* species ranges from Least Concern (LC) to Vulnerable (VU) (Table 1). *T. temminckii* and *T. satyra* are classified as LC, with *T. temminckii* having an estimated global population exceeding 100,000 individuals, while recent assessments suggest that *T. satyra* may also number in the high tens to hundreds of thousands of individuals. In contrast, *T. caboti*, *T. blythii*, and *T. melanocephalus* are listed as VU and have substantially smaller populations, with *T. caboti* estimated at approximately 3500–15,000 individuals, *T. blythii* at 3500–15,000 individuals, and *T. melanocephalus* at about 3200–9500 mature individuals [28]. There is an urgent need to quantify the distribution dynamics of *Tragopan* birds to guide conservation actions. Furthermore, existing research on the *Tragopan* birds remains limited to single climate scenario analysis, fails to integrate future climate–vegetation interactions, and mostly focuses on individual species, with few studies exploring the impacts of multiple factors on the distribution of *Tragopan* birds [27,29]. Therefore, this study used the Maxent model to assess the impact of climate change on the geographical distribution of *Tragopan* birds. This study focuses on the following questions: (1) What are the main factors affecting the distribution of *Tragopan* birds? (2) How will the future suitable habitats of *Tragopan* birds change? (3) Will *Tragopan* birds be able to successfully colonize their future suitable habitats? Unlike previous studies that focused on single *Tragopan* species or only climatic variables, this study provides three novel contributions: (1) simultaneous modeling of all five *Tragopan* species in China under a consistent framework; (2) integration of multiple anthropogenic factors (land use, human footprint) and four SSP climate scenarios; and (3) quantitative comparison of centroid migration velocity versus dispersal velocity to assess whether *Tragopan* birds can track future habitat shifts.

**Table 1:** List of *Tragopan* species present globally and their status.

Scientific Name	IUCN Redlist Status	CITES	Population Trend	Habitat Type	Source
*Tragopan temminckii*	Least concern	[28]	Decreasing	Forest, Shrubland	https://www.iucnredlist.org/species/22679169/264164899. Accessed on 10 June 2025.
*Tragopan satyra*	Least concern	[28]	Decreasing	Forest	https://www.iucnredlist.org/species/22679157/254103308. Accessed on 10 June 2025.
*Tragopan caboti*	Vulnerable	[28]	Decreasing	Forest	https://www.iucnredlist.org/species/22679172/177687593. Accessed on 10 June 2025.
*Tragopan melanocephalus*	Vulnerable	[28]	Decreasing	Forest	https://www.iucnredlist.org/species/22679147/177694929. Accessed on 10 June 2025.
*Tragopan blythii*	Vulnerable	[28]	Decreasing	Forest	https://www.iucnredlist.org/species/22679163/177682428. Accessed on 10 June 2025.

Note: Source form IUCN redlist. https://www.iucnredlist.org. Accessed on 10 June 2025.

## 2. Materials and Methods

### 2.1. Data Collecting

Geographical distribution data of *Tragopan* birds in China were collected from three sources. First, the Global Biodiversity Information Facility (GBIF) is the world’s largest network and data infrastructure, funded by governments worldwide, with the aim of providing open access to data on all forms of life on Earth. Accordingly, 1072 occurrence records of *Tragopan* birds in China, spanning from the early 2000s to the end of 2023, were downloaded from GBIF (https://doi.org/10.15468/dL.czgz2k. Accessed on 25 November 2024). Second, a total of 327 bird watching records were gathered from websites including the China Bird Watching Record Center (https://www.birdreport.cn/. Accessed on 25 November 2024) and eBird (https://ebird.org/. Accessed on 25 November 2024). Third, 55 records were collected from the published literature. To minimize location inaccuracies, Google Earth (http://earth.google.com. Accessed on 25 November 2024) was utilized to verify locations, and records with inaccurate information or duplicates were removed. Ultimately, 526 valid distribution records of *Tragopan* birds in China were obtained (Table S1). These observations were not repeated measurements of the same individuals within the same season but rather included both adult individuals with reproductive territoriality and migratory juveniles.

### 2.2. Environmental Variables

When considering the complex interactions among bioclimatic variables, it is essential to select appropriate variables (i.e., those relevant to species ecology) to predict the climatically suitable areas for a species [30]. Therefore, 27 environmental factor datasets were chosen as candidate variables for the Maxent model (Table 2 and Table S2). Among the 27 environmental variables, climate variables (temperature, precipitation, etc.) were obtained for current, LGM, and future periods from the respective climate layers. Non-climatic variables (e.g., HFI, NDVI, vegetation type, land use) were held constant across all time periods at their current values (representing the recent past or present) because historical and future projections for these layers are either unavailable or highly uncertain at the spatial and temporal resolution required. This approach assumes that non-climatic factors do not change over time, which is a simplification; we therefore emphasize that our results primarily reflect climate-driven habitat suitability changes, while land use and human influence are treated as static background constraints. And 19 were bioclimatic factor data [31], including temperature-related factors (Bio 01–11) and precipitation-related factors (Bio 12–19); one hydrological factor, specifically the distance to major water sources (Dis_water); two vegetation factors, including the Normalized Difference Vegetation Index (NDVI) and vegetation type (VEG); three topographic factors, namely Elevation, Aspect, and Slope; one human disturbance factor, i.e., the Human Footprint Index (HFI); and one socioeconomic factor, specifically land use type (CLCD).

In addition, data for the Last Glacial Maximum (LGM) were obtained from the CCSM4 model (CMIP5) [32], which has been shown to perform well in simulating climate over China. Future climate data (SSP126, SSP245, SSP370, SSP585) were sourced from CMIP6 models via WorldClim (version 2.1). To ensure comparability across time periods, we applied a delta method bias correction using the current climate as a baseline, following standard protocols for combining CMIP5 paleoclimate and CMIP6 future projections [33]. While differences in model generations introduce some uncertainty, this approach is widely used in species distribution modeling when paleoclimate data from newer CMIP6 are not available for the LGM. Under each scenario, data for three time periods were included: 2041–2060 (2050s), 2061–2080 (2070s), and 2081–2100 (2090s).

All environmental variables were standardized to the same coordinate system (GCS_WGS_1984) using ArcGIS 10.8 and resampled to a common spatial resolution of 1 km (≈30 arc-seconds). For continuous variables (e.g., temperature, precipitation, HFI, NDVI), we applied bilinear interpolation; for categorical variables (e.g., vegetation type, land use class), we used nearest neighbor resampling to preserve original class values. The choice of 1 km resolution represents a compromise that retains regional-scale patterns while accommodating the coarsest input layers (originally 2.5′ climate data, ≈4.5 km, were downscaled using bilinear interpolation to 1 km). Then, they were subjected to operations such as projection and conversion to convert them into the ASCII format required by the Maxent model for subsequent analysis.

**Table 2:** Variables used in the model prediction.

Variables	Indicators	Data Sources	Resolution
Bioclimatic variables	Bio 01–Bio 11 (Temperature) Bio 12–Bio 19 (precipitation)	WorldClim (https://www.worldclim.org. Accessed on 25 November 2024)	2.5′
Distance to water source	Bio 20 (Dis_water)	Resource and Environmental Science Data Center of the Chinese Academy of Sciences (https://www.resdc.cn/. Accessed on 25 November 2024)	2.5′
Topographic dataset	Bio 21 (DEM) Bio 22 (Slope) Bio 23 (Aspect)	WorldClim (https://www.worldclim.org. Accessed on 25 November 2024)	2.5
Vegetation dataset	Bio 24 (NDVI) Bio 25 (VEG)	Resource and Environmental Science Data Center of the Chinese Academy of Sciences (https://www.resdc.cn/. Accessed on 25 November 2024)	30 m 1 km
Socioeconomic dataset	Bio 26 (HFI)	Socioeconomic Data and Applications Center (https://sedac.ciesin.columbia.edu/. Accessed on 25 November 2024)	1 km
Land use	Bio 27 (CLCD)	The 30 mannual land cover dataset and its dynamics in China from 1990 to 2019 [34]	30 m

Note: Slope and aspect were derived from the SRTM (Shuttle Radar Topography Mission) digital elevation model (DEM) at 30 arc-second (~1 km) resolution. The SRTM DEM was downloaded from [source: the USGS EarthExplorer or CGIAR-CSI], and slope (in degrees) and aspect (converted to eastness and northness) were calculated using the Surface Analyst tool in ArcGIS 10.8.

### 2.3. Data Cleaning

Before modeling, the downloaded distribution records were cleaned to improve model performance and avoid overfitting [35,36]. Records with identical coordinates from the same or different sources were treated as duplicates, and only one was retained. Records were checked using Google Earth (http://earth.google.com. Accessed on 25 November 2024). Records falling in obvious error locations (e.g., water bodies, cities far from known habitats) or with coordinate uncertainty > 1 km were removed. Records with missing or implausible coordinates (e.g., latitude/longitude = 0) were also excluded. All occurrence records used in the final dataset (*n* = 315) are from the period 2000–2023. This ensures consistency with the current climate data (WorldClim 2.1, 1970–2000) and reduces bias from historical distribution changes.

ENMTools.pl was used to reduce spatial clustering of occurrence records by retaining only one record per 5 km × 5 km grid cell (i.e., a minimum distance of 5 km between retained records). This distance corresponds approximately to the spatial resolution of the climate layers (2.5 arc-minutes, ≈4.5–5 km at the study latitudes), ensuring that no two records fall within the same climate grid cell [37,38]. Finally, 315 thinned occurrence records were used to simulate the potential distribution of *Tragopan* birds in China (Figure 1f, Table S1). These records were saved in CSV format as required by Maxent 3.4.1 for subsequent analysis.

**Figure 2:**
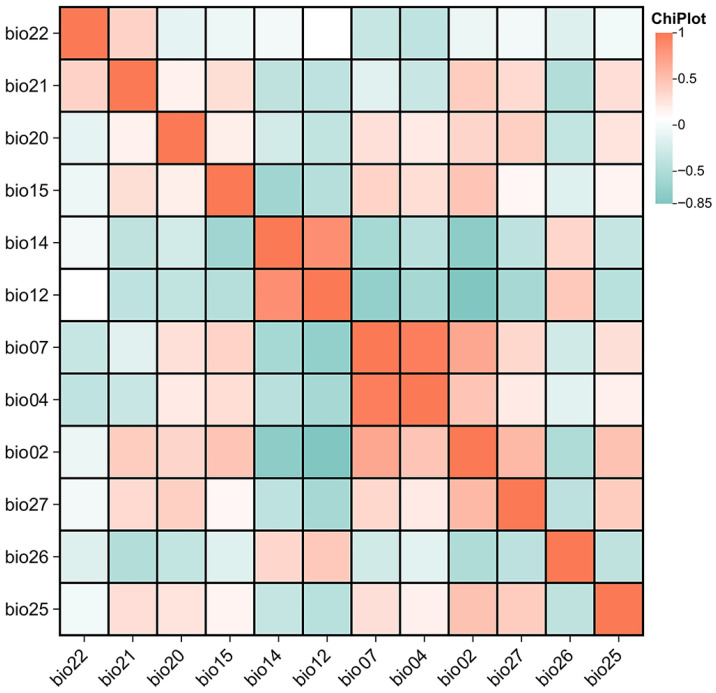
Correlation coefficient matrix of 12 environment variables.

To avoid model overfitting caused by multicollinearity among environmental data [39], the Pearson product-moment correlation coefficient was used to test the collinearity of the 27 environmental factors (Figure S1). Variables with a pairwise Pearson correlation coefficient greater than 0.8 were identified as highly collinear (Table S2). In such cases, we retained the variable that was considered more ecologically relevant to Tragopan habitat requirements (e.g., retaining mean annual temperature over maximum temperature of warmest month) based on the published literature and species natural history [40]. Ultimately, 12 environmental factors with statistical and biological significance were selected for modeling (Figure 2), including mean diurnal temperature range (Bio 02), temperature seasonality (Bio 04), temperature annual range (Bio 07), annual precipitation (Bio 12), precipitation of driest month (Bio 14), precipitation seasonality (Bio 15), distance to water source (Dis_water/Bio 20), elevation (Bio 21), slope (Bio 22), vegetation type (VEG/Bio 25), human footprint index (HFI/Bio 26), and China Land Cover Dataset (CLCD/Bio 27).

### 2.4. Maxent Model Development and Evaluation

To avoid overfitting due to multicollinearity, we calculated the Pearson correlation coefficients for the 27 candidate variables (Figure S1). Pairs with |r| > 0.8 were considered highly collinear [2]; from each pair, the variable with a lower contribution in preliminary Maxent runs was removed. Variables with near-zero contribution (e.g., NDVI, Bio 24) were also excluded. This process retained 12 environmental variables for final modeling (Figure 2). Continuous environmental variables were designated as “Continuous”, while discrete environmental variables were labeled as “Categorical”. The option “Create response curves” was checked; a random 25% of the distribution points were selected as the test set, and the remaining 75% as the training set [41]. To ensure model stability, the number of replicates was set to 10, with “Bootstrap” selected as the replication type [42]. The Jackknife test was used to evaluate the contribution of each environmental factor to the distribution of *Tragopan* birds [43]. All other Maxent parameters were kept at default values, including auto feature classes, regularization multiplier = 1, 10,000 background points, convergence threshold = 10^−5^, and maximum iterations = 500. Other parameters were set to default, and results were output in ASCII format.

The area under the Receiver Operating Characteristic (ROC) curve, known as the AUC (Area Under Curve) value, was used to assess the accuracy of the model predictions [44]. The AUC value ranges from 0 to 1: a higher AUC indicates that the species distribution deviates more from a random distribution, reflecting a stronger correlation between environmental variables and the model and thus more reliable predictions [45]. The evaluation criteria for AUC are as follows: 0.5–0.6 (fail), 0.6–0.7 (poor), 0.7–0.8 (fair), 0.8–0.9 (good), and 0.9–1.0 (excellent).

The key environmental factors influencing *Tragopan* bird distribution were identified based on factor contribution rates and Jackknife analysis results [46]. ArcGIS 10.8 was used for visualizing Maxent output results and classifying habitat suitability levels [47,48]. Using the reclassification function in ArcToolbox, potential suitable habitats were divided into four grades: unsuitable habitat (0.0–0.2), least suitable habitat (0.2–0.4), moderately suitable habitat (0.4–0.6), and highly suitable habitat (>0.6). The total suitable area is the sum of low, moderate, and high suitability regions.

The “Distribution Change Between Binary SDMs” tool in SDM Tools was employed to calculate the area of habitat changes (contraction, expansion, and stability) across different periods [49,50]. The values were defined as follows: 0 (unsuitable habitat), −1 (expanded habitat), 1 (stable habitat), and 2 (contracted habitat). Based on the changes in potential suitable habitats of *Tragopan* birds across the aforementioned periods, the “Centroid Changes Lines” tool in SDM Tools (version 2.6) was used to calculate the variation in the geometric centroid and migration distance of potential suitable habitats across different periods (consistent results across 10 model replicates) [51], thereby analyzing the overall change trend of potential suitable areas for *Tragopan* birds over time.

### 2.5. Bioclimatic Velocity and Dispersal Velocity

Bioclimatic velocity refers to the rate at which species are exposed to climate change; translating physical climatic space into bioclimatic space helps inform conservation decisions within a given time frame [52]. Given that populations at the core of a species’ distribution may be more resilient to the impacts of adverse environmental changes than those at the distribution margins, core populations are expected to persist longer than marginal populations [2,53]. Therefore, the centroid migration velocity of *Tragopan* populations under future climate change was calculated. Dispersal velocity refers to the distance a species spreads over a given period, which depends on the species’ body mass, diet type, generation time, and variations in displacement distance [2,54]. Dispersal events of *Tragopan* birds were calculated based on the positive allometric relationship between adult body mass (M) and median dispersal distance (D), as shown in Equation (1). The adult body mass of *Tragopan* birds was set to 0.92–1.80 kg [55].

DV = 2.1 × M^0.18^/T (1)

Note: M = adult body mass (kg), T = generation time (years), DV = dispersal velocity (km/year).

## 3. Results

### 3.1. Model Performance and Variable Contribution

Maxent modeling was performed using the filtered distribution records and environmental factors. The average AUC value across 10 modeling runs was 0.961, with an average test AUC value of 0.949 and an average training AUC value of 0.961 (Figure S2). All prediction results reached the “Excellent” level, indicating that the model predictions can accurately reflect the habitat distribution of *Tragopan* birds in China.

**Figure 3:**
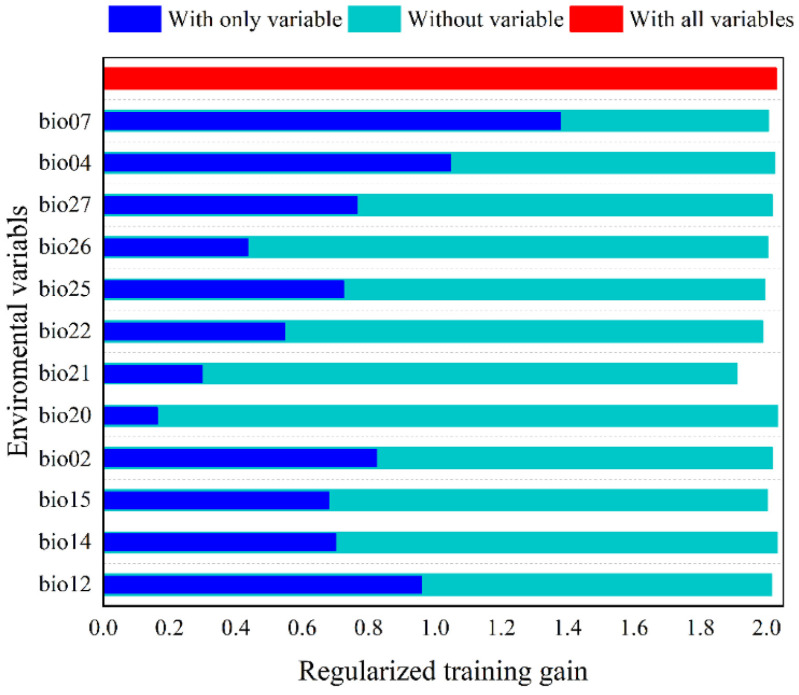
Jackknife text of the importance of environment variables in Maxent.

The relative contribution (e.g., percentage contributions and permutation importance) of each environmental factor was evaluated using the Jackknife test (Figure 3, Table S2). The top six environmental factors ranked by percentage contribution and permutation importance were identified as the dominant factors influencing the geographical distribution of *Tragopan* birds. These six factors contributed 79.3% to the model’s prediction of *Tragopan* birds distribution, of which land use type (CLCD/Bio 27) had the highest contribution (25.00%), followed by temperature annual range (Bio 07, 23.8%), annual precipitation (Bio 12, 16.70%), elevation (Bio 21, 6.70%), vegetation type (Bio 25, 5.00%), and human footprint index (Bio 26, 2.10%). In terms of permutation importance ranking, temperature annual range (Bio 07, 24.30%) was the most critical environmental factor affecting the potential distribution of *Tragopan* birds, followed by elevation (Bio 21, 17.20%), annual precipitation (Bio 12, 10.20%), human footprint index (Bio 26, 7.70%), vegetation type (Bio 25, 2.30%), and land use type (CLCD/Bio 27, 1.30%). According to the Jackknife test results, when variables were used individually, temperature annual range (Bio 07) was the most important environmental factor, with the highest regularized training gain (1.38), test gain (1.45), and AUC (0.91). Bio 04, Bio 12, and Bio 02 also exhibited relatively high normalized gain values, highlighting their significant contributions to model performance. However, when these factors were excluded from the model, the gain decreased significantly, indicating that the potential distribution probability of *Tragopan* birds is relatively sensitive to these factors.

### 3.2. Response of the Tragopan Birds to Critical Environmental Factors

**Figure 4:**
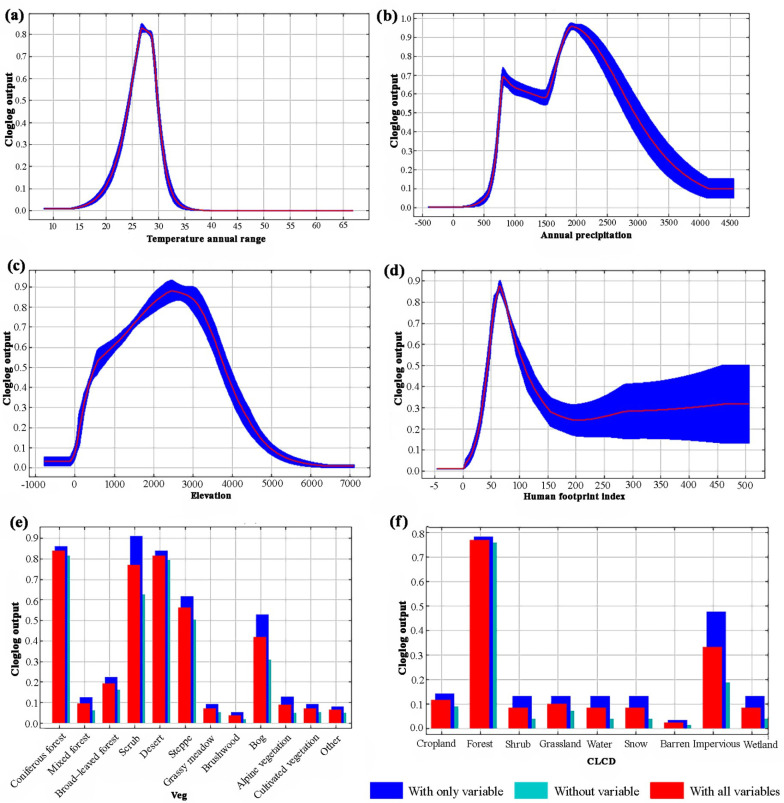
Response curves of habitat suitability predicted by the MaxEnt model for *Tragopan* birds based on key environmental variables. (**a**) Response curve for temperature annual range. (**b**) Response curve for annual precipitation. (**c**) Response curve for elevation. (**d**) Response curve for human footprint index. (**e**) Habitat suitability across vegetation types (Veg). (**f**) Habitat suitability across land cover types (CLCD).

Species response curves describe the relationship between environmental factors and the probability of species presence, revealing a species’ preferences and tolerance for habitats [56]. The response ranges of dominant environmental factors to potential habitats were observed from their respective species response curves (Figure 4). Results showed that *Tragopan* birds had the strongest dependence on coniferous forests and shrubs (Bio 25), with a presence probability of up to 75%, mainly determined by food availability and breeding habits. The presence probability of *Tragopan* birds exhibited an inverted “V”-shaped response curve with elevation (Bio 21) and human footprint index (Bio 26). This indicates that habitats with moderate and high elevations (1500–3300 m) and moderate human activity intensity (5–10) are more suitable for *Tragopan* birds’ survival. When the presence probability exceeded 0.7, an annual temperature range of 24–30 °C was relatively suitable, corresponding to a higher probability of *Tragopan* bird occurrence. Annual precipitation (Bio 12) represents precipitation variation, and the optimal annual precipitation range for *Tragopan* birds was 1500–2600 mm. Regarding land use type (CLCD/Bio 27), woodlands (mixed forests, evergreen coniferous forests) served as the primary habitats for *Tragopan* birds. In summary, the above results indicate that moist habitats with moderate and high elevations, small temperature variations, and moderate human activity intensity (such as woodlands and shrubs) are the main habitats of *Tragopan* birds.

### 3.3. The Past and Current Suitable Habitat of the Tragopan Birds

**Figure 5:**
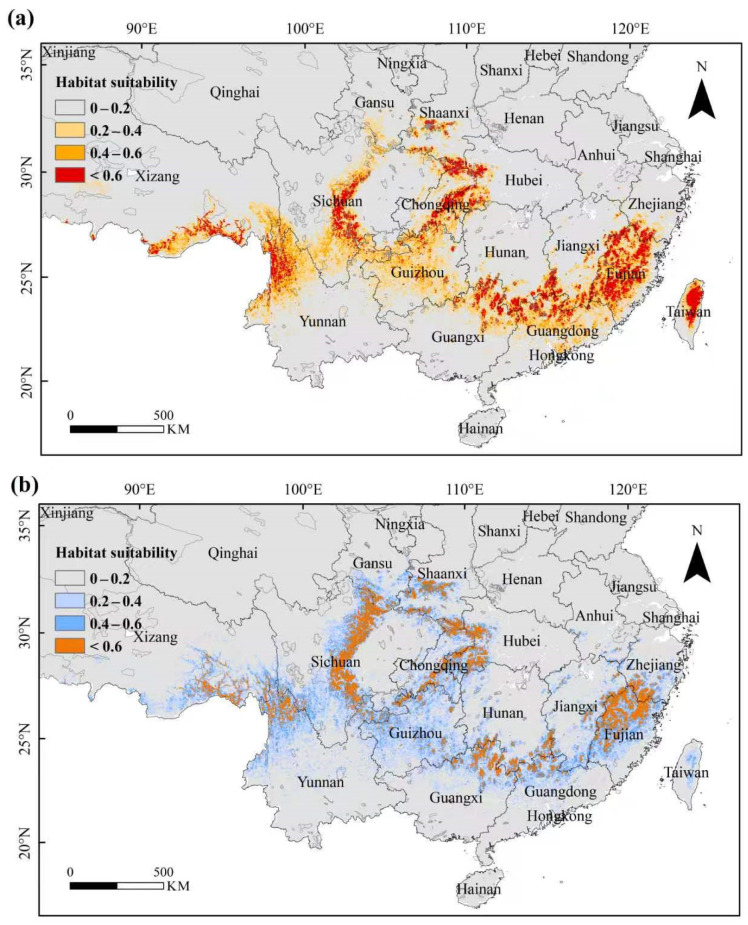
The suitable habitats of *Tragopan* birds in different periods. (**a**) LGM: Last Glacial Maximum. (**b**) Current.

During the Last Glacial Maximum (LGM), the total suitable habitat area of *Tragopan* birds was 80.05 × 10^4^ km^2^, covering most areas of southeastern and southwestern China. The highly suitable habitats (17.21 × 10^4^ km^2^) were mainly distributed in regions such as Fujian, Sichuan, Yunnan, and Tibet in China, accounting for 21.50% of the total suitable habitat area; the moderately suitable habitats (19.90 × 10^4^ km^2^) were primarily located in areas including Yunnan, Sichuan, Fujian, and Guizhou, representing 24.86% of the total suitable habitat area; and the least suitable habitats (42.94 × 10^4^ km^2^) were mainly distributed in regions like Sichuan, Guizhou, Yunnan, and Guangdong, making up 53.64% of the total suitable habitat area (Figure 5a).

The Maxent model was used to identify the potential suitable habitats of *Tragopan* birds under current climatic conditions. The total suitable habitat area of *Tragopan* birds was 97.83 × 10^4^ km^2^, mainly distributed in the warm and humid regions south of the Yellow River in China, showing an overall trend of expanding distribution from south to north. The highly suitable habitats were primarily located in the southwest, central, and southeast regions of China, with an area of 20.91 × 10^4^ km^2^, accounting for 21.37% of the total potential suitable habitats; the moderately suitable habitats were concentrated in the southwest and southeast regions, covering 24.18 × 10^4^ km^2^ (24.72% of the total); and the least suitable habitats were mainly distributed in the southwest, central, and southeast regions, with an area of 52.74 × 10^4^ km^2^, representing 53.91% of the total potential suitable habitats (Figure 5b).

ArcGIS 10.8 was used to classify the potential suitable habitats of *Tragopan* birds in China, and the suitable habitat areas in 34 provincial level administrative regions were obtained (the top 8 provincial level administrative regions are shown) (Table S3). The results indicated that Sichuan Province had the largest suitable habitat area (17.72 × 10^4^ km^2^), followed by Guizhou, Yunnan, Fujian, Tibet, Guangxi, Jiangxi, and Hunan Provinces/Autonomous Regions. In contrast, regions such as Liaoning, Jiangsu, Hainan, Beijing, Tianjin, and Shanghai were unsuitable for *Tragopan* birds, with almost no distribution.

In addition, a further analysis was conducted on the proportion of highly suitable habitats in the total suitable habitats of each administrative region. The proportions in descending order were Hubei, Fujian, Shaanxi, Zhejiang, Sichuan, Chongqing, Guangxi, and Hunan. Hubei Province had the highest proportion (36.38%), making it the optimal distribution area for *Tragopan* birds. Regions such as Henan and Ningxia lacked highly suitable habitats, indicating that their environmental conditions are unsuitable for *Tragopan* survival.

### 3.4. Predicted Future Suitable Habitat of the Tragopan Birds

**Figure 6:**
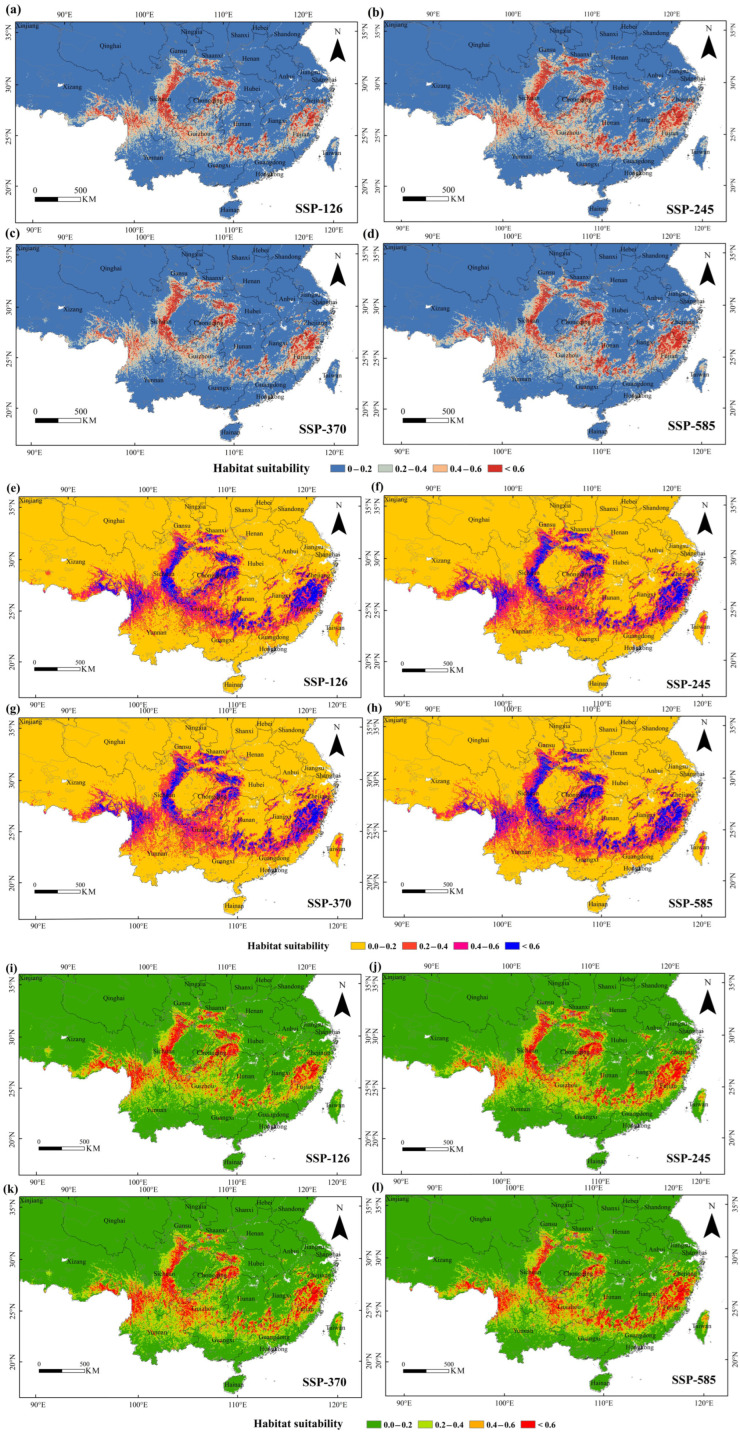
The suitable habitats of *Tragopan* birds in different periods. (**a**–**d**) 2041–2060. (**e**–**h**) 2061–2080. (**i**–**l**) 2081–2100.

Based on the four climate scenarios (SSP126, SSP245, SSP370, and SSP585) proposed by the IPCC, the potential suitable habitats of *Tragopan* birds were predicted for the periods 2041–2060 (2050s), 2061–2080 (2070s), and 2081–2100 (2090s) (Figure 6, Figure S3, Table S4). The results showed that the locations and areas of various suitable habitats of *Tragopan* birds changed to different degrees, but they remained mainly distributed in southwestern and southeastern China.

In 2050, the comparison of highly suitable habitats under different scenarios was as follows: SSP245 > SSP585 > SSP370 > SSP126. The highly suitable habitat area under the SSP245 scenario was the largest (24.87 × 10^4^ km^2^), increasing by 15.92% compared with the current climatic conditions. The increase in the total area of potential suitable habitats was mainly attributed to the expansion of least suitable habitats (at the junction of Hunan and Guizhou, as well as in Fujian, Zhejiang, Gansu, Yunnan, and Guangxi) and moderately suitable habitats (in Shaanxi, Hunan, Yunnan, Zhejiang, Anhui, Fujian, and Guizhou) (Figure 6a–d).

In 2070, the total area of highly suitable habitats under the SSP585 scenario was the largest (24.48 × 10^4^ km^2^), with an increase of 17.00%, and the most significant area change among all scenarios. The increase in total area was primarily due to the conversion to moderately suitable habitats in Tibet, Yunnan, Sichuan, Guizhou, Guangxi, Zhejiang, Fujian, Hunan, and Hubei, as well as the conversion to least suitable habitats at the junctions of Tibet, Yunnan, Sichuan, Hunan, Guangxi, Zhejiang, Taiwan, Tibet, and Xinjiang (Figure 6e–h).

In 2090, the area of highly suitable habitats under the SSP585 scenario was 27.34 × 10^4^ km^2^, increasing by 30.75%. The increase in total area was mainly driven by the expansion of moderately suitable habitats at the junction of Yunnan and Guizhou, as well as in Guangxi, Guangdong, Fujian, and Zhejiang, with significant contributions from least suitable habitats in Yunnan, Guangxi, Guangdong, and Zhejiang. Overall, under future climate scenarios, the concentration of potential suitable habitats for *Tragopan* birds will increase, and most regions will show a significant expansion trend (Figure 6i–l).

### 3.5. Change in Suitable Habitat of the Tragopan Birds

**Figure 7:**
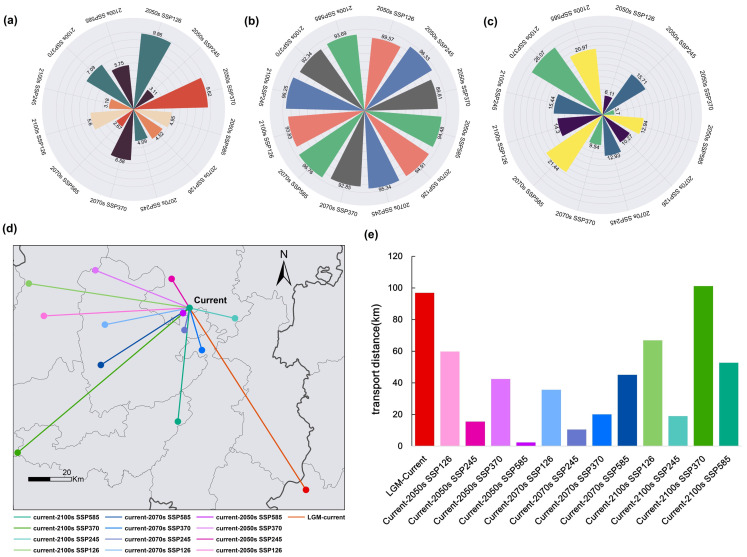
Centroid shifts of *Tragopan* birds based on future climate scenarios. (**a**) Expansion area. (**b**) Stability area. (**c**) Construction area. (**d**) Centroid shifts. (**e**) Migration distance.

The relative changes in potential future species distribution were estimated by comparing differences in suitable habitats across the current, past, and future periods. This analysis revealed that with the passage of time, the continuous expansion of the suitable habitat range and the reduction in fragmentation of *Tragopan* birds are associated with climate change (Figure 7a–c, Table 3).

From Table 3, we can observe that under the SSP126 scenario, compared with the current period, the 2050s saw the largest loss of habitat area (3.75 × 10^4^ km^2^), while the 2090s witnessed the largest gain in habitat area (8.70 × 10^4^ km^2^). Under the SSP245 scenario, habitat changes showed a trend of first decreasing and then increasing: the 2090s had the largest gain (12.26 × 10^4^ km^2^), followed by the 2050s (11.99 × 10^4^ km^2^) and the 2070s (8.84 × 10^4^ km^2^). SSP370 represents the impact of extreme land use and land cover changes on climate; under this scenario, 5.92 × 10^4^ km^2^ of suitable habitat was lost in the 2050s, but the suitable habitat range increased by 18.98 × 10^4^ km^2^ in the 2090s. Under the SSP585 scenario, the suitable habitat area increased by 18.77 × 10^4^ km^2^ in the 2070s, with increases of 7.99 × 10^4^ km^2^ and 15.22 × 10^4^ km^2^ in the 2050s and 2090s, respectively (Table 3).

Compared with the current period, new habitats in the 2050s will be mainly distributed in southwestern China, specifically in Yunnan, Guangxi, Guizhou, Sichuan, Hunan, and Zhejiang (Figure 6). Most lost habitats will be located in coastal areas (Fujian, Guangdong, and Taiwan) as well as at the junctions of Chongqing, Guangxi, and Guangdong. In the 2070s, the main expansion areas will be in southwestern China (Yunnan, Guangxi, and Sichuan), while the main contraction areas will be in the southeastern coastal regions (Fujian and Guangdong) (Figure 6). In the 2090s, Yunnan will have the largest expansion area, followed by Guizhou and Guangxi; Jiangxi and Chongqing will experience the largest contraction ranges (Figure 6).

In summary, under all four future climate scenarios, the total suitable habitat range of *Tragopan* birds shows an overall increasing trend over time. SSP585 exhibits the largest increase amplitude, followed by SSP245, SSP370, and SSP126.

### 3.6. Centroid Shifts of Tragopan Birds in Future Climatic Scenario

Centroid migration can intuitively demonstrate the change trajectory of the suitable habitat areas for *Tragopan* birds (Figure 7d,e). During the Last Glacial Maximum (LGM), the centroid was located in Tongren City, Guizhou Province (27.52° N, 107.91° E), and migrated 96.83 km northwestward to its position in the current period. In the current period, the centroid is situated in Zheng’an County, Zunyi City, Guizhou Province (28.28° N, 107.43° E).

From the current period to the 2050s, the centroids under the four different emission scenarios all migrate within the boundaries of Guizhou Province. Based on these four scenarios, 16 migration routes from the current period to the future were mapped, as detailed below:

Under the SSP126 emission scenario: The centroid migrates 59.84 km southwestward from its current position to the 2050s centroid (28.25° N, 106.82° E), then moves a further 35.61 km southwestward to the 2070s centroid (28.21° N, 107.07° E) and finally migrates 66.84 km northwestward to the 2090s centroid (28.38° N, 106.76° E).

Under the SSP245 emission scenario: The centroid migrates 15.48 km northwestward from its current position to the 2050s centroid (28.40° N, 107.36° E), then shifts 10.46 km southwestward to the 2070s centroid (28.18° N, 107.40° E) and ultimately moves 19.00 km southeastward to the 2090s centroid (28.23° N, 107.61° E).

Under the SSP370 emission scenario: The centroid migrates 42.41 km northwestward from its current position to the 2050s centroid (28.43° N, 107.03° E), then travels 20.07 km southeastward to the 2070s centroid (28.10° N, 107.47° E) and finally moves 101.12 km southwestward to the 2090s centroid (27.67° N, 106.70° E).

Under the SSP585 emission scenario: The centroid migrates southwestward in all periods: first moving 2.36 km to the 2050s centroid (28.25° N, 107.40° E), then shifting 45.07 km to the 2070s centroid (28.04° N, 107.05° E), finally migrating 52.7 km to the 2090s centroid (27.80° N, 107.38° E).

### 3.7. Biological Velocity and Dispersal Velocity of Tragopan Birds

Climate change and human activities are expected to cause the core range of *Tragopan* birds to shift by 59.84 km (SSP126), 35.61 km (SSP126), and 66.84 km (SSP126), respectively (Table S5). The average speed of *Tragopan* birds is approximately 1 km per year, whether calculated as biological speed or dispersal speed. A comparison between the dispersal velocity and biological velocity of *Tragopan* birds reveals that despite the gradual expansion of the predicted habitat area, *Tragopan* birds are unable to expand into new suitable habitat regions.

## 4. Discussion

Climate change and human activities have exposed wildlife to greater survival challenges, making the conservation of animal diversity (especially rare and protected species) one of the most pressing scientific issues to address [2,10]. We used the Maximum Entropy Model (Maxent) to map the climatic niches of these five *Tragopan* birds. These models not only predicted the current climatically suitable areas for *Tragopan* birds but also forecasted potential changes in their climatic niches and distributions under intensifying warming scenarios across three time scales. These results showed that under the SSP585 scenario, relatively favorable conditions were predicted for the 2050s, 2070s, and 2090s, with the total suitable habitat range expected to show an overall increasing trend over time. Due to climate warming, *Tragopan* birds are gradually migrating toward lower latitudes and elevations. We found that land use is a key determinant influencing the habitat suitability of *Tragopan* birds. Notably, despite the predicted gradual expansion of *Tragopan* habitat area, the biological velocity of *Tragopan* birds fails to keep pace with the shift in their core range. This indicates that *Tragopan* birds are unable to expand into new suitable habitat areas.

The distribution of *Tragopan* birds is influenced by human activities and climate change. This study found that changes in land use, temperature, precipitation and elevation could all have a significant impact on the distribution of *Tragopan* birds. These results are consistent with the findings of Cui et al. [57], Liu et al. [58], Zhao et al. [27], and Jameel et al. [59]. Specifically, this study revealed that both human-induced factors (particularly land use) and climate variables (temperature annual range, annual precipitation) jointly determine habitat suitability [60]. In our results, land use (CLCD) had the highest percent contribution (25.0%), followed closely by temperature annual range (23.8%) and annual precipitation (16.7%). Therefore, we consider land use and climate as co-dominant drivers, with their relative importance varying across space and time [1,59].

This study found that temperature annual range (Bio 07) and annual precipitation (Bio 12) play important roles in the distribution of *Tragopan* birds. The annual temperature range of 24–30 °C and an optimal annual precipitation of 1500–2600 mm are relatively suitable for the survival and life activities of *Tragopan* birds. This indicates that *Tragopan* birds prefer areas with moderate temperatures and humid habitats, and traces of their activities are more likely to be found near rivers or streams. These results are in line with the studies of Liu et al. [58] and Zhao et al. [27]. Temperatures above or below the optimal range will affect the final distribution of *Tragopan* birds. If temperatures are too low, female birds will spend more time and energy on incubation, reducing their foraging time and harming their survival. Additionally, low temperatures are unfavorable for the survival of young birds [60]. When temperatures are too high, heat stress may occur, causing physiological dysfunction [61]. Precipitation affects *Tragopan* birds’ habitat selection primarily by influencing their food sources. *Tragopan* birds mainly feed on plant fruits or seeds in autumn and winter [60]. Precipitation affects the growth of these trees and the diversity of *Tragopan* birds’ food sources, thereby influencing their final distribution [7,62].

Elevation is also a key factor affecting the distribution of *Tragopan* birds. This study found that *Tragopan* birds are most commonly distributed in the upper part of moderate and high mountains to alpine regions, particularly around 2500 m. This is consistent with the findings of Cui et al. [57] and Jameel et al. [59]. *Tragopan* birds often inhabit temperate or subtropical mountainous areas and have developed a certain adaptive range to climate factors such as temperature and humidity during long-term evolution. Elevation is the most direct regulatory factor of mountain microclimates [59]. In low-elevation areas, excessively high temperatures in summer easily cause heat stress; in high-elevation areas, severe cold in winter may affect survival due to food scarcity and cold damage [29,61]. High-elevation areas usually have high air humidity and high annual precipitation, and the humid environment helps prevent *Tragopan* birds’ skin from drying and cracking. At the same time, high humidity maintains the abundance of food sources (e.g., seeds, insect larvae) [27,60]. The intensity of human activities (e.g., agricultural reclamation, logging, tourism development) decreases with increasing elevation, effectively avoiding extensive human disturbance to *Tragopan* birds [59].

In the different future climate scenarios, the suitable habitats of many species face the risk of reduction or even loss [2,10,14]. For example, Wang et al. [10] found that the habitat area of red-crowned cranes (*Grus japonensis*) will continue to shrink in the future, habitat suitability will decrease significantly, and habitat connectivity between different regions will also deteriorate noticeably. Ye et al. [2] also found that by 2050, the suitable habitat area of two langur species is expected to decrease by 45–47%. However, in this study, we found that the suitable habitat area of *Tragopan* birds will expand to varying degrees under several future climate change scenarios. This is consistent with the results of Jameel et al. [59] and Xia et al. [24]. Jameel et al. [59] found that under future climate change scenarios, the habitat suitability of the *T. melanocephalus* may improve. Xia et al. [24] also found that the suitable habitat area of the crested ibis will increase significantly under future climate change scenarios.

In this study, the increase in the suitable habitat area of *Tragopan* birds may be attributed to the expansion of low and moderately suitability habitats. Currently, *Tragopan* birds are mainly distributed in Sichuan, Yunnan, Guizhou, Fujian, and other regions. Although the suitable habitats will change to varying degrees under different shared socioeconomic pathways (SSP126, SSP245, SSP370, SSP585) in the 2050s, 2070s, and 2090s, they will still be mainly distributed in southwestern and southeastern China. Additionally, the concentration of suitable habitats will increase, and most regions will show a significant expansion trend. These areas are mostly mountainous, with elevations mostly within a suitable range, and they also have diverse microhabitats required for *Tragopan* birds’ survival. They possess rich vegetation types (e.g., evergreen broadleaved forests, coniferous–broadleaved mixed forests), and sufficient precipitation ensures the vigorous growth of vegetation in these areas. This not only provides a good habitat but also offers abundant food (e.g., fruits, seeds, insects) for *Tragopan* birds [27,29,57,60]. Most areas in southwestern and southeastern China have subtropical or tropical monsoon climates, which are warm and humid, conducive to the survival and reproduction of *Tragopan* birds [60,63]. Some areas in Sichuan, Yunnan, Guizhou, Chongqing, Fujian, and other regions are geographically remote with inconvenient transportation, resulting in less human impact. This allows the habitats of *Tragopan* birds to remain relatively intact [59,60,63].

Threatened species face significant challenges in adapting to rapid environmental changes caused by human activities and climate change [64]. If a species can successfully migrate to expanded suitable habitat areas, it will be beneficial for the species’ survival and reproduction, thereby promoting population growth. If a species cannot colonize newly formed habitats, it may experience a significant reduction in distribution range and population size and may eventually face regional extinction [18,65]. In this study, the suitable habitat area of *Tragopan* birds will increase under different future climate scenarios, and their dispersal velocity approaches their biological velocity, but the species still faces significant challenges in expanding into their optimal habitats. This is not only because *Tragopan* birds are poor fliers with weak dispersal capabilities but also because the intensification of human activities and the increase in migration barriers formed by human infrastructure (highways, villages, farmlands, cities) severely restricts the ability of *Tragopan* birds to track changes in suitable habitats [27,59,66]. Several sources of uncertainty should be considered when interpreting our predictions. First, future climate projections under SSP scenarios vary substantially. SSP126 represents a low-emission, sustainable pathway, while SSP585 assumes continued high emissions without mitigation. The marked differences in predicted habitat area between these scenarios highlight the sensitivity of Tragopan distributions to the magnitude of future warming. Second, our Maxent models assume that the relationship between environmental variables and species occurrence remains constant over time (i.e., niche conservatism), which may not hold under rapid climate change. Third, presence-only data suffer from sampling bias, as occurrence records are concentrated in easily accessible areas (e.g., nature reserves). Although we applied spatial thinning to reduce clustering, some bias likely remains. Fourth, we assumed non-climatic variables (land use, human footprint, vegetation) remain static in future projections, which may underestimate the combined effects of land use change and climate change. Future studies should incorporate dynamic land use projections when available. Based on our findings, we suggest that establishing ecological corridors between current and future predicted suitable habitats could be a useful conservation strategy, although this inference requires further empirical testing. We also recommend setting up new protected areas in regions where future climate change may create new suitable habitats for *Tragopan* birds.

This study has several limitations that should be acknowledged. First, we merged all five Tragopan species into a single occurrence set to model genus-level responses. While these species share broadly similar macroecological niches, this approach may obscure interspecific differences in climate sensitivity and dispersal ability. Future studies should model each species separately to refine predictions. Second, our centroid migration velocity is not equivalent to standard bioclimatic velocity (climate velocity), which tracks the movement of climate isotherms. Therefore, our comparison with dispersal velocity is exploratory rather than definitive. Third, the dispersal velocity formula relies on allometric scaling with adult body mass; the coefficients (2.1 and 0.18) are derived from Sutherland et al. [54] for birds, but uncertainty in body mass estimates (0.92–1.80 kg) and generation time (assumed 5 years) may affect the calculated values. Fourth, our Maxent models used default parameters without tuning (e.g., regularization multiplier = 1), which may lead to overfitting. Future work should employ model tuning (e.g., ENMeval) to optimize complexity. Finally, we assumed non-climatic variables remain unchanged in future scenarios due to data unavailability; dynamic projections of land use and human footprint would improve realism.

## 5. Conclusions

*Tragopan* birds are mainly distributed in southwestern China (e.g., Sichuan, Guizhou) and southeastern coastal regions (e.g., Zhejiang, Fujian), with particularly favorable habitat conditions in Sichuan, Chongqing, and Fujian. Under most future climate scenarios (SSP126, SSP245, SSP370), their suitable habitat is projected to contract; however, under the high-emission SSP585 scenario, the total suitable habitat range shows an overall increasing trend over time. Centroid analysis indicates a projected shift toward lower latitudes and elevations under climate warming. Environmental factor analysis identifies both human-induced factors (particularly land use) and climate variables (temperature annual range, annual precipitation) as joint determinants of habitat suitability. Comparison between the range dispersal velocity (1.04 km/yr) and centroid migration velocity (1.08 km/yr) indicates that *Tragopan* birds may be capable of tracking future habitat shifts. However, constrained by realistic environmental perturbations including human activities, climate change and urbanization, the migration of *Tragopan* birds is largely limited, which may hinder their effective tracking of future habitat dynamics. Notably, these findings remain exploratory at this stage. Five species may exhibit differentiated climatic preferences and distribution dynamics. Future studies are recommended to model each species individually and conduct interspecific comparisons.

## Figures and Tables

**Table 3:** Changes in suitability areas for *Tragopan* birds over time under different SSP periods.

Periods	Area/(×10^4^ km^2^)			
	Expansion	Stability	Construction	Change
LGM-Current	27.91	71.53	10.00	17.91
Current-2050s SSP126	6.11	89.57	9.86	−3.75
Current-2050s SSP245	15.71	96.33	3.11	11.99
Current-2050s SSP370	3.7	89.81	9.62	−5.92
Current-2050s SSP585	12.94	94.48	4.95	7.99
Current-2070s SSP126	10.27	94.91	4.52	5.75
Current-2070s SSP245	12.93	95.34	4.09	8.84
Current-2070s SSP370	9.54	92.85	6.58	2.92
Current-2070s SSP585	21.44	96.76	2.67	18.77
Current-2100s SSP126	14.3	93.83	5.6	8.7
Current-2100s SSP245	15.44	96.25	3.18	12.26
Current-2100s SSP370	26.07	92.34	7.09	18.98
Current-2100s SSP585	20.97	93.69	5.75	15.22

## Data Availability

All data generated or analyzed during this study are included in this published article and its Supplementary Information Files.

## Supplementary Materials

The following supporting information can be downloaded at: https://www.mdpi.com/article/10.3390/biology15090713/s1, Table S1: Distribution records of *Tragopan* species used for species distribution modeling in China. Table S2: Percent contribution and permutation importance of environmental variables in the Maxent model. Table S3: Areal extent of suitable habitats for *Tragopan* species across Chinese provinces (×10^4^ km^2^). Table S4: Projected changes in the area of suitable habitats for *Tragopan* species under future climate scenarios (2050s, 2070s, and 2090s) (×10^4^ km^2^). Table S5: Area of suitable habitats within China’s national nature reserves across different periods (×10^4^ km^2^). Figure S1: Correlation coefficient matrix of 27 environment variables. Figure S2: ROC curves of the Maxent model. Figure S3: The habitat area of *Tragopan* species in China under four Shared Socioeconomic Pathways (SSP126, SSP245, SSP370, SSP585) in the years 2050s (a–d), 2070s (e–h), and 2090s (i–l). Subpanels a, e, i: SSP126; b, f, j: SSP245; c, g, k: SSP370; d, h, l: SSP585.

## References

[1] Urban M.C. (2015). Accelerating extinction risk from climate change. Science.

[2] Ye X.L., Garber P.A., Li M., Zhao X.M. (2024). Climate and anthropogenic activities threaten two langur species irrespective of their range size. Divers. Distrib..

[3] IPCC (2021). Climate Change 2021: The Physical Science Basis.

[4] Smith S.J., Edmonds J., Harlin C.A., Mundra A., Calvin K. (2015). Near-term acceleration in the rate of temperature change. Nat. Clim. Change.

[5] Chiang F., Mazdiyasni O., AghaKouchak A. (2021). Evidence of anthropogenic impacts on global drought frequency, duration, and intensity. Nat. Commun..

[6] Wu S.J., Luo M., Lau G.N.C., Zhang W., Wang L., Liu Z., Lin L.J., Wang Y.J., Ge E.R., Li J.F. (2025). Rapid flips between warm and cold extremes in a warming world. Nat. Commun..

[7] Pinkert S., Farwig N., Kawahara A.Y., Jetz W. (2025). Global hotspots of butterfly diversity are threatened in a warming world. Nat. Ecol. Evol..

[8] Zhang M., Yuan X., Zeng Z.Z., Pan M., Wu P.L., Xiao J.F., Keenan T.F. (2025). A pronounced decline in northern vegetation resistance to flash droughts from 2001 to 2022. Nat. Commun..

[9] Scheffers B.R., De Meester L., Bridge T.C.L., Hoffmann A.A., Pandolfi J.M., Corlett R.T., Butchart S.H.M., Pearce-Kelly P., Kovacs K.M., Dudgeon D. (2016). The broad footprint of climate change from genes to biomes to people. Science.

[10] Wang G., Wang C., Guo Z., Dai L., Wu Y., Liu H., Li Y., Chen H., Zhang Y., Zhao Y. (2020). Integrating Maxent model and landscape ecology theory for studying spatiotemporal dynamics of habitat: Suggestions for conservation of endangered Red-crowned crane. Ecol. Indic..

[11] McGill B. (2015). Land use matters. Nature.

[12] Beissinger S.R., MacLean S.A., Iknayan K.J., de Valpine P. (2023). Concordant and opposing effects of climate and land-use change on avian assemblages in California’s most transformed landscapes. Sci. Adv..

[13] Haddad N.M., Brudvig L.A., Clobert J., Davies K.F., Gonzalez A., Holt R.D., Lovejoy T.E., Sexton J.O., Austin M.P., Collins C.D. (2015). Habitat fragmentation and its lasting impact on Earth’s ecosystems. Sci. Adv..

[14] Sarkar D., Talukdar G. (2023). Predicting the impact of future climate changes and range-shifts of Indian hornbills (family: Bucerotidae). Ecol. Inform..

[15] Horváth Z., Ptacnik R., Vad C.F., Chase J.M. (2019). Habitat loss over six decades accelerates regional and local biodiversity loss via changing landscape connectance. Ecol. Lett..

[16] Tayleur C., Caplat P., Massimino D., Johnston A., Jonzén N., Smith H.G., Lindström Å. (2015). Swedish birds are tracking temperature but not rainfall: Evidence from a decade of abundance changes. Glob. Ecol. Biogeogr..

[17] Poloczanska E.S., Brown C.J., Sydeman W.J., Kiessling W., Schoeman D.S., Moore P.J., Brander K., Bruno J.F., Buckley L.B., Burrows M.T. (2013). Global imprint of climate change on marine life. Nat. Clim. Change.

[18] Sales L., Ribeiro B.R., Chapman C.A., Loyola R. (2020). Multiple dimensions of climate change on the distribution of Amazon primates. Perspect. Ecol. Conser..

[19] Elith J., Leathwick J.R. (2009). Species distribution models: Ecological explanation and prediction across space and time. Annu. Rev. Ecol. Evol. Syst..

[20] Kaky E., Nolan V., Alatawi A., Gilbert F. (2020). A comparison between Ensemble and MaxEnt species distribution modelling approaches for conservation: A case study with Egyptian medicinal plants. Ecol. Inform..

[21] Wisz M.S., Hijmans R.J., Li J., Peterson A.T., Graham C.H., Guisan A., Distribut N.P.S. (2008). Effects of sample size on the performance of species distribution models. Divers. Distrib..

[22] Jeong A., Kim M., Lee S. (2024). Analysis of priority conservation areas using habitat quality models and MaxEnt models. Animals.

[23] Singh M. (2020). Evaluating the impact of future climate and forest cover change on the ability of Southeast (SE) Asia’s protected areas to provide coverage to the habitats of threatened avian species. Ecol. Indic..

[24] Zhuoyi X., Jie S., Haiwei Y., Fanhua K. (2023). Temporal and spatial patterns of habitat of *Nipponia nippon* in China under the back-ground of climate change. Chin. J. Appl. Ecol..

[25] BirdLife International (2022). State of the World’s Birds 2022.

[26] Li X.Y., Clinton N., Si Y.L., Liao J.S., Liang L., Gong P. (2015). Projected impacts of climate change on protected birds and nature reserves in China. Sci. Bull..

[27] Zhao Z.H., Yu S.S., Gong X.F., Chen Z.Q., Wang Y.F., Zhao J.L., Wei L., Fan X.L., Lin Z.H. (2023). Effects of climate change on potential suitable habitat of *Tragopan caboti*. Acta Ecol. Sin..

[28] IUCN (2025). The IUCN Red List of Threatened Species. Version 2025-2. https://www.iucnredlist.org.

[29] Pang L.F., Yu T.L. (2023). Prediction of potential habitat for *Tragopan caboti* based on MaxEnt model. J. Guangxi Norm. Univ. Nat. Sci. Ed..

[30] Mathur M., Mathur P., Purohit H. (2023). Ecological niche modelling of a critically endangered species *Commiphora wightii* (Arn.) Bhandari using bioclimatic and non-bioclimatic variables. Ecol. Process..

[31] Fick S.E., Hijmans R.J. (2017). WorldClim 2: New 1-km spatial resolution climate surfaces for global land areas. Int. J. Climatol..

[32] Gent P.R., Danabasoglu G., Donner L.J., Holland M.M., Hunke E.C., Jayne S.R., Lawrence D.M., Neale R.B., Rasch P.J., Vertenstein M. (2011). The community climate system model version 4. J. Clim..

[33] Wu T.W., Lu Y.X., Fang Y.J., Xin X.G., Li L., Li W.P., Jie W.H., Zhang J., Liu Y.M., Zhang L. (2019). The Beijing climate center climate system model (BCC-CSM): The main progress from CMIP5 to CMIP6. Geosci. Model Dev..

[34] Yang J., Huang X. (2021). The 30 m annual land cover dataset and its dynamics in China from 1990 to 2019. Earth Syst. Sci. Data.

[35] Boria R.A., Olson L.E., Goodman S.M., Anderson R.P. (2014). Spatial filtering to reduce sampling bias can improve the performance of ecological niche models. Ecol. Model..

[36] Li Y.C., Li M.Y., Li C., Liu Z.Z. (2020). Optimized Maxent Model Predictions of Climate Change Impacts on the Suitable Distribution of *Cunninghamia lanceolata* in China. Forests.

[37] Beck J., Böller M., Erhardt A., Schwanghart W. (2014). Spatial bias in the GBIF database and its effect on modeling species’ geographic distributions. Ecol. Inform..

[38] Shi X.D., Wang J.W., Zhang L., Chen S.X., Zhao A.L., Ning X.D., Fan G.R., Wu N.S., Zhang L., Wang Z.D. (2023). Prediction of the potentially suitable areas of *Litsea cubeba* in China based on future climate change using the optimized MaxEnt model. Ecol. Indic..

[39] Radosavljevic A., Anderson R.P. (2014). Making better Maxent models of species distributions: Complexity, overfitting and evaluation. J. Biogeogr..

[40] Feng X., Park D.S., Liang Y., Pandey R., Papes M. (2019). Collinearity in ecological niche modeling: Confusions and challenges. Ecol. Evol..

[41] Pramanik M., Diwakar A.K., Dash P., Szabo S., Pal I. (2021). Conservation planning of cash crops species (*Garcinia gummigutta*) under current and future climate in the Western Ghats, India. Environ. Dev. Sustain..

[42] Jiang R.P., Zou M., Qin Y., Tan G.D., Huang S.P., Quan H.G., Zhou J.Y., Liao H. (2022). Modeling of the potential geographical distribution of three *Fritillaria* species under climate change. Front. Plant Sci..

[43] Wang F., Yuan X.Z., Sun Y.J., Liu Y.H. (2024). Species distribution modeling based on MaxEnt to inform biodiversity conservation in the Central Urban Area of Chongqing Municipality. Ecol. Indic..

[44] Lobo J.M., Jiménez-Valverde A., Real R. (2008). AUC: A misleading measure of the performance of predictive distribution models. Glob. Ecol. Biogeogr..

[45] Zhao R.F., Wang S.J., Chen S.Y. (2024). Predicting the potential habitat suitability of Saussurea species in China under future climate scenarios using the optimized Maximum Entropy (MaxEnt) model. J. Clean. Prod..

[46] Tan Y.F., Tan X.H., Yu Y.P., Zeng X.P., Xie X.Q., Dong Z.T., Wei Y.L., Song J.Y., Li W.X., Liang F. (2024). Climate change threatens *Barringtonia racemosa*: Conservation insights from a MaxEnt Model. Diversity.

[47] Ab Lah N.Z., Yusop Z., Hashim M., Salim J.M., Numata S. (2021). Predicting the habitat suitability of *Melaleuca cajuputi* based on the MaxEnt species distribution model. Forests.

[48] Xie C.P., Huang B.Y., Jim C.Y., Han W.D., Liu D.W. (2021). Predicting differential habitat suitability of *Rhodomyrtus tomentosa* under current and future climate scenarios in China. For. Ecol. Manag..

[49] Brown J.L., Bennett J.R., French C.M. (2017). SDMtoolbox 2.0: The next generation Python-based GIS toolkit for landscape genetic, biogeographic and species distribution model analyses. PeerJ.

[50] Gebrewahid Y., Abrehe S., Meresa E., Eyasu G., Abay K., Gebreab G., Kidanemariam K., Adissu G., Abreha G., Darcha G. (2020). Current and future predicting potential areas of *Oxytenanthera abyssinica* (A. Richard) using MaxEnt model under climate change in Northern Ethiopia. Ecol. Process..

[51] Hao Y.L., Dong P.B., Wang L.Y., Ke X., Hao X.F., He G., Chen Y., Guo F.X. (2024). Predicting the potential distribution of *Hypericum perforatum* under climate change scenarios using a Maximum Entropy Model. Biology.

[52] Serra-Diaz J.M., Franklin J., Ninyerola M., Davis F.W., Syphard A.D., Regan H.M., Ikegami M. (2014). Bioclimatic velocity: The pace of species exposure to climate change. Divers. Distrib..

[53] Channell R., Lomolino M.V. (2000). Trajectories to extinction: Spatial dynamics of the contraction of geographical ranges. J. Biogeogr..

[54] Sutherland G.D., Harestad A.S., Price K., Lertzman K.P. (2000). Scaling of natal dispersal distances in terrestrial birds and mammals. Conserv. Ecol..

[55] MacKinnon J., Phillipps K., He F. (2000). A Field Guide to the Birds of China.

[56] Savic A., Dmitrovic D., Glöer P., Pesic V. (2020). Assessing environmental response of gastropod species in karst springs: What species response curves say us about niche characteristic and extinction risk?. Biodivers. Conserv..

[57] Cui P., Kang M.J., Deng W.H. (2008). Foraging habitat selection by sympatric Temminck’s tragopan and blood pheasant during breeding season in southwestern China. Bio. Sci..

[58] Liu X.B., Wei W., Zheng X.G., Zhao K.H., He S.W., Zhou W.L. (2017). Activity rhythms of golden pheasant (*Chrysolophus pictus*) and satyr tragopan (*Tragopan temmminckii*) revealed by infrared-triggered cameras. Chin. J. Zool..

[59] Jameel M.A., Nadeem M.S., Haq S.M., Mubeen I., Shabbir A., Aslam S., Ahmad R., Gaafar A.R.Z., Al-Munqedhi B.M.A., Bussmann R.W. (2023). Shifts in the distribution range and niche dynamics of the globally threatened western tragopan (*Tragopan melanocephalus*) due to climate change and human population pressure. Biology.

[60] Zheng G.M. (2015). Pheasants in China.

[61] Vandana G.D., Sejian V., Lees A.M., Pragna P., Silpa M.V., Maloney S.K. (2021). Heat stress and poultry production: Impact and amelioration. Int. J. Biometeorol..

[62] Kim J., Lee D.K., Kim H.G. (2020). Suitable trees for urban landscapes in the Republic of Korea under climate change. Landsc. Urban Plan..

[63] Zhao F., Zhang B.P., Zhu L.Q., Yao Y.H., Cui Y.P., Liu J.J. (2019). Spectra structures of altitudinal belts and their significance for determining the boundary between warm temperate and subtropical zones in the Qinling-Daba Mountains. Acta Geogr. Sin..

[64] Foden W.B., Young B.E., Akçakaya H.R., Garcia R.A., Hoffmann A.A., Stein B.A., Thomas C.D., Wheatley C.J., Bickford D., Carr J.A. (2019). Climate change vulnerability assessment of species. WIREs Clim. Change.

[65] Schloss C.A., Nuñez T.A., Lawler J.J. (2012). Dispersal will limit ability of mammals to track climate change in the Western Hemisphere. Proc. Natl. Acad. Sci. USA.

[66] Wang W., Qiao Y., Li S., Pan W., Yao M. (2017). Low genetic diversity and strong population structure shaped by anthropogenic habitat fragmentation in a critically endangered primate, *Trachypithecus leucocephalus*. Heredity.

