# Supplementary material for: Impacts of Anthropogenic Activities and Climate Change on the Distribution Ranges of Five Tragopan Birds in China
Source: Biology (Basel). 2026 Apr 30;15(9):713. doi: 10.3390/biology15090713 (PMC13162768; doi:10.3390/biology15090713)
Supplement: Supplementary file 1 [file biology-15-00713-s001.zip › Supplementary materials.pdf]

Table S1 Distribution records of *Tragopan* species used for species distribution modeling in China.

Scientific name	Birdreport、ebird	GBIF	Literature	Removing	Total number
Tragopan temminckii	172	611	0	289	175
Tragopan satyra	26	30	2	21	10
Tragopan caboti	129	431	28	200	114
Tragopan melanocephalus	0	0	3	3	3
Tragopan blythii	0	0	22	13	13

Table S2 Percent contribution and permutation importance of environmental variables in the Maxent model.

Indicators	Description	Percent contribution	Permutation importance
Bio 01	Annual mean temperature	0.6	9.1
Bio 02	Mean diurnal temperature range	1.8	1.5
Bio 03	Isothermality	0.4	0.7
Bio 04	Temperature seasonality	4.4	32.2
Bio 05	Maximum temperature of warmest month	0.3	0.3
Bio 06	Minimum temperature of coldest month	0.8	4.0
Bio 07	Temperature annual range	21.8	10.8
Bio 08	Mean temperature of wettest quarter	2.2	0.3
Bio 09	Mean temperature of driest quarter	0.1	0.1
Bio 10	Mean temperature of warmest quarter	0.4	3.0
Bio 11	Mean temperature of coldest quarter	1.6	0.2
Bio 12	Annual precipitation	11.8	0.6
Bio 13	Precipitation of wettest month	0.4	3.7
Bio 14	Precipitation of driest month	2.1	0.8
Bio 15	Precipitation seasonality	1.4	2.1
Bio 16	Precipitation of wettest quarter	0.4	1.7
Bio 17	Precipitation of driest quarter	1.0	0.9
Bio 18	Precipitation of warmest quarter	0.3	5.4
Bio 19	Precipitation of coldest quarter	1.5	8.9
Bio 20	Distance to water source	0.2	0.5
Bio 21	Elevation	5.1	4.8
Bio 22	Slope	10.8	2.9
Bio 23	Aspect	0.8	0.3
Bio 24	Normalized Difference Vegetation Index	0	0
Bio 25	Vegetation type	3.0	0.4
Bio 26	Human footprint index	1.9	3.5
Bio 27	China Land Cover Datase	25.1	1.2

Table S3 Areal extent of suitable habitats for *Tragopan* species across Chinese provinces (×10^4 km^2).

Region	Unsuitable habitat	Lowly suitable habitat	Moderately suitable habitat	Highly suitable habitat	Total suitable habitat
Sichuan	30.84	8.70	4.08	4.94	17.72
Guizhou	5.84	8.19	2.73	0.84	11.76
Yunnan	27.26	6.08	2.86	1.37	10.31
Fujian	2.96	3.00	2.70	3.03	8.74
Xizang	110.37	5.24	1.95	1.29	8.48
Guangxi	17.43	3.41	1.20	1.34	5.96
Jiangxi	11.25	2.93	1.36	0.88	5.18
Hunan	15.83	2.94	1.06	1.13	5.13
Chongqing	3.49	2.32	1.10	1.27	4.70
Guangdong	12.62	2.67	1.06	0.57	4.30
Hubei	14.20	1.53	1.16	1.54	4.23
Shanxi	16.63	1.64	1.04	1.25	3.93
Zhejiang	6.66	1.33	0.80	0.88	3.02
Gansu	39.65	1.63	0.71	0.51	2.85
Anhui	13.18	0.47	0.16	0.07	0.70
Taiwan	2.52	0.51	0.18	0.00	0.70
Henan	16.38	0.11	0.01	0.00	0.11
Ningxia	5.18	0.02	0.00	0.00	0.02
Xinjiang	161.42	0.00	0.00	0.00	0.00
Neimenggu	113.35	0.00	0.00	0.00	0.00
Qinghai	69.67	0.00	0.00	0.00	0.00
Heilongjiang	44.37	0.00	0.00	0.00	0.00
Jilin	18.85	0.00	0.00	0.00	0.00
Hebei	18.67	0.00	0.00	0.00	0.00
Shaanxi	15.68	0.00	0.00	0.00	0.00
Shandong	14.98	0.00	0.00	0.00	0.00
Liaoning	14.17	0.00	0.00	0.00	0.00
Jiangsu	9.75	0.00	0.00	0.00	0.00
Hainan	2.95	0.00	0.00	0.00	0.00
Beijing	1.64	0.00	0.00	0.00	0.00
Tianjin	1.13	0.00	0.00	0.00	0.00
Shanghai	0.57	0.00	0.00	0.00	0.00
Hongkong	0.11	0.00	0.00	0.00	0.00
Macao	0.0029	0.00	0.00	0.00	0.00
Total	839.58	52.74	24.18	20.91	97.83

Table S4 Projected changes in the area of suitable habitats for *Tragopan* species under future climate scenarios (2050s, 2070s, and 2090s) (×10^4 km^2).

Period	LGM	Current	2050s				2070s				2100s			
			SSP126	SSP245	SSP370	SSP585	SSP126	SSP245	SSP370	SSP585	SSP126	SSP245	SSP370	SSP585
Unsuitable habitat	587.62	839.78	841.68	825.28	843.87	829.65	834.10	831.07	836.98	821.18	831.23	827.65	820.83	821.17
Lowly suitable habitat	42.94	52.76	53.82	58.09	50.70	56.38	52.92	56.15	54.29	57.49	53.48	54.28	58.63	57.49
Moderately suitable habitat	19.90	24.19	24.30	29.28	24.50	27.31	26.00	26.79	25.07	30.49	27.78	28.80	30.84	29.85
Highly suitable habitat	17.21	20.91	17.71	24.87	18.44	24.19	24.62	23.64	21.31	24.48	25.15	26.84	27.33	27.34

Table S5 Area of suitable habitats within China's national nature reserves across different periods (×10^4 km^2).

Period	Unsuitable habitat	Lowly suitable habitat	Moderately suitable habitat	Highly suitable habitat	Total suitable habitat
LGM	91.98	1.77	1.12	1.62	4.51
Current	90.07	2.38	1.47	2.57	6.42
2050s	90.03	2.36	1.51	2.58	6.45
2070s	90.06	2.35	1.48	2.61	6.43
2090s	90.01	2.25	1.51	2.73	6.49

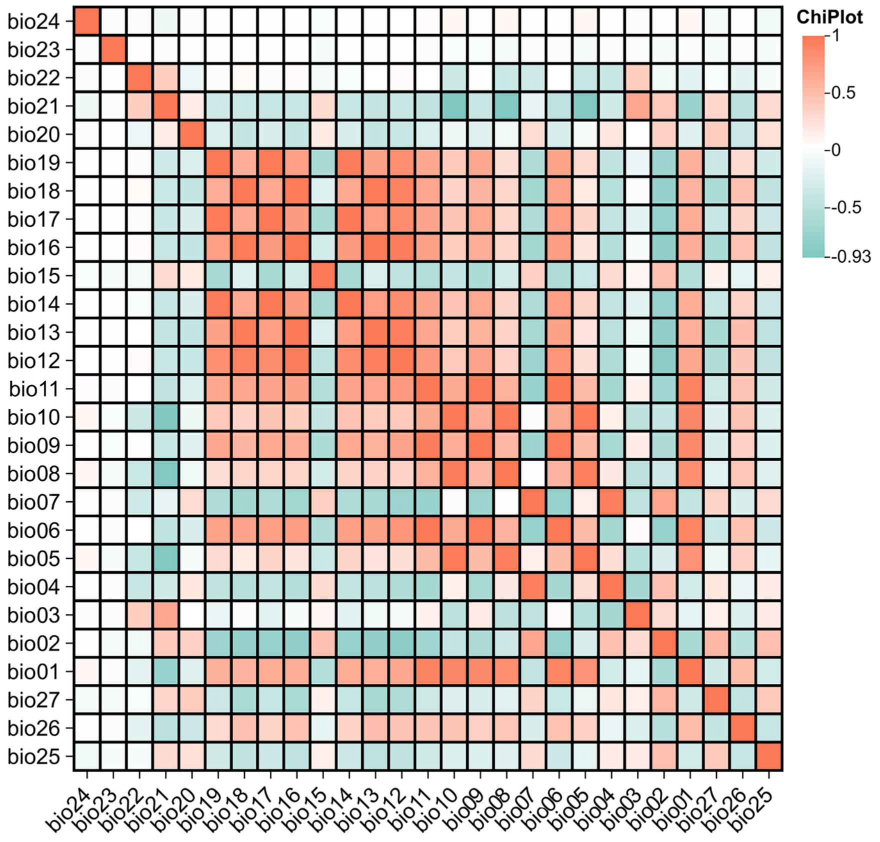

Figure S1 Correlation coefficient matrix of 27 environment variables.

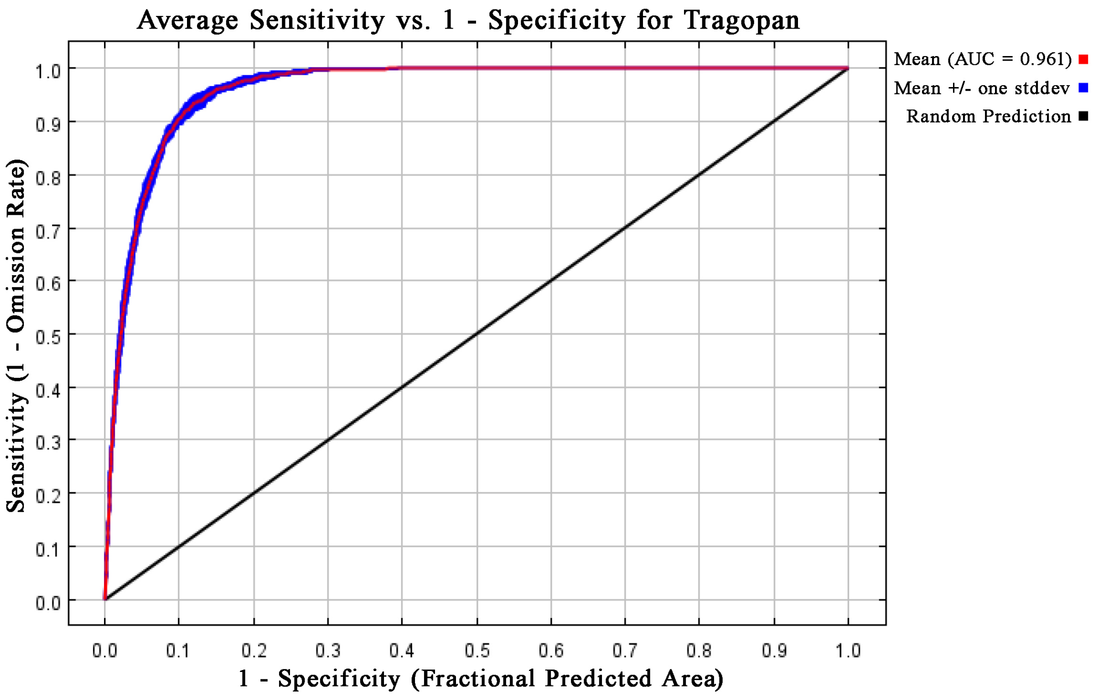

Figure S2 ROC curves of the Maxent model.

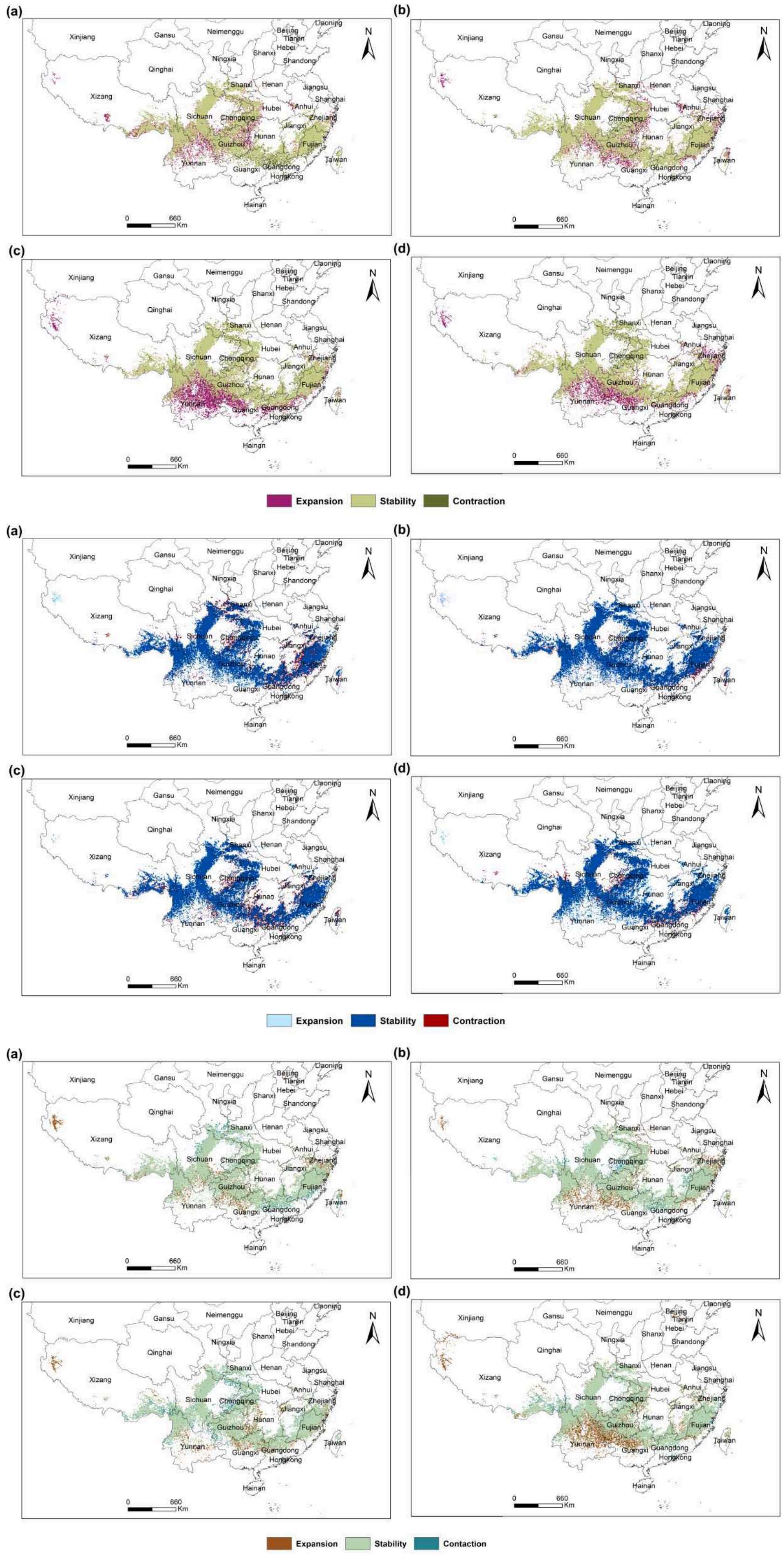

Figure S3 The habitat area of *Tragopan* species in China under four Shared Socioeconomic Pathways (SSP126, SSP245, SSP370, SSP585) in the years 2050s (a-d), 2070s (e-h), and 2090s (i-l). Subpanels a, e, i: SSP126; b, f, j: SSP245; c, g, k: SSP370; d, h, l: SSP585.
